# Updating the Food-Based Dietary Guidelines for the Spanish Population: The Spanish Society of Community Nutrition (SENC) Proposal

**DOI:** 10.3390/nu11112675

**Published:** 2019-11-05

**Authors:** Javier Aranceta-Bartrina, Teresa Partearroyo, Ana M. López-Sobaler, Rosa M. Ortega, Gregorio Varela-Moreiras, Lluis Serra-Majem, Carmen Pérez-Rodrigo, The Collaborative Group for the Dietary Guidelines for the Spanish Population (SENC)

**Affiliations:** 1 Department of Food Sciences and Physiology, University of Navarra, Pamplona, 31009 Navarra, Spain; 2 CIBEROBN, Biomedical Research Networking Center for Physiopathology of Obesity and Nutrition, Carlos III Health Institute, 28029 Madrid, Spain; 3 Research Institute of Biomedical and Health Sciences (IUIBS), University of Las Palmas de Gran Canaria, 35016 Las Palmas, Spain; 4 Department of Physiology, Faculty of Medicine, University of the Basque Country (UPV/EHU), 48940 Leioa, Vizcaya, Spain; 5 Departamento de Ciencias Farmacéuticas y de la Salud, Facultad de Farmacia, Universidad San Pablo-CEU, CEU Universities, Urbanización Montepríncipe, 28925 Alcorcón, Madrid, Spain; 6 Departamento de Nutrición y Ciencia de los Alimentos, Facultad de Farmacia, Universidad Complutense de Madrid, 28040 Madrid, Spain; 7 Spanish Nutrition Foundation (FEN), 28010 Madrid, Spain; 8 Fundación para la Investigación Nutricional (FIN), 08029 Barcelona, Spain

**Keywords:** dietary guidelines, physical activity, food habits, Mediterranean diet, health, sustainability, primary healthcare, community health

## Abstract

Diet-related risk factors and physical inactivity are among the leading risk factors for disability and are responsible for a large proportion of the burden of chronic non-communicable diseases. Food-based dietary guidelines (FBDGs) are useful tools for nutrition policies and public health strategies to promote healthier eating and physical activity. In this paper, we discuss the process followed in developing the dietary guidelines for the Spanish population by the Spanish Society of Community Nutrition (SENC) and further explain the collaboration with primary healthcare practitioners as presented in the context of the NUTRIMAD 2018 international congress of SENC. From a health in all policies approach, SENC convened a group of experts in nutrition and public health to review the evidence on diet-health, nutrient intake and food consumption in the Spanish population, as well as food preparation, determinants and impact of diet on environmental sustainability. The collaborative group drafted the document and designed the graphic icon, which was then subject to a consultation process, discussion, and qualitative evaluation. Next, a collaborative group was established to plan a dissemination strategy, involving delegates from all the primary healthcare scientific societies in Spain. A product of this collaboration was the release of an attractive, easy-to-understand publication.

## 1. Introduction

Non-communicable diseases (NCDs), such as cardiovascular diseases, cancer, chronic respiratory diseases, and diabetes are major causes of disability, ill health, and premature death in the European Union (EU) and globally, resulting in considerable social and economic costs [1]. In the EU, it has been estimated that, as the leading cause of mortality, most healthcare expenses are due to the fact of NCDs, costing EU economies 0.8% of GDP annually [2]. 

According to the Food and Agriculture Organization of the United Nations (FAO) definition, “Sustainable diets are those diets with low environmental impact, which contribute to food and nutrition security and to healthy life for present and future generations” [3]. Hence, sustainable diets are protective and respectful of biodiversity and ecosystems, culturally acceptable, accessible, economically fair, and affordable. In addition, such diets are nutritionally adequate, safe, and healthy; while optimizing natural and human resources. Within that framework, the sustainability of diets is not only focused on nutrition and the environment, but beyond those, it relates to economic and sociocultural dimensions.

Food choices that form dietary patterns affect the demand for production of agricultural products, but agricultural systems are part of a larger food system that includes producing, processing, food manufacturing, and distribution to consumers. Sustainable food systems are critical for achieving healthy, sustainable diets [4]. Likewise, Dernini et al. [5] have made important efforts to characterize the multiple dimensions and benefits of the Mediterranean diet as a sustainable diet and developed a framework (Med Diet 4.0) representing four sustainability benefits of the Mediterranean diet: major health and nutrition benefits, low environmental impact and richness in biodiversity, high sociocultural food values, and positive local economic returns.

The United Nations and the World Health Organization (WHO) defined nine global targets for NCDs to reach by 2025 [6]. In line with this, Sustainable Development Goal 3.4 aims to reduce premature mortality from non-communicable diseases by one-third and promote mental health and well-being by 2030 [7]. Diet-related risk factors and physical inactivity are among the leading risk factors for disability and are responsible for a large proportion of the burden of chronic non-communicable diseases globally [8]. Providing evidence-based guidance for healthier dietary patterns and lifestyles can play an important role for public health [9]. Integrated approaches across sectors and policy fields are required to face the challenge of NCDs with a focus on prevention [6]. Food-based dietary guidelines (FBDG) are useful tools for nutrition policies and public health strategies to promote healthier eating and physical activity [10,11]. They serve as an approach to increase awareness and provide the public, healthcare professionals, policy makers, and others with evidence-based practical dietary and lifestyle advice using an understandable language. Food-based dietary guidelines are often combined with a visual representation to help consumers’ understanding [10,11]. In this paper, we discuss the process followed to develop the dietary guidelines for the Spanish population by the Spanish Society of Community Nutrition (SENC, Appendix A) and further explain the collaboration with primary healthcare practitioners as discussed in the context of the NUTRIMAD 2018 international congress of the Spanish Society for Community Nutrition.

## 2. Food-Based Dietary Guidelines

Food-based dietary guidelines have been defined as science-based recommendations in the form of guidelines for healthy eating [10,11]. They are easy-to-understand documents intended for consumers as well as policy makers, healthcare professionals, the food industry, and educational purposes [10,11]. In order to be significant for the target audience, FBDGs have to be appropriate for the region or country, culturally acceptable, easy to adopt, and include practical advice [10,11,12,13]. Relevant issues about the target population, such as prevailing food habits and dietary patterns, nutrient sources and health-disease patterns have to be considered when developing FBDGs [10,11,12,13]. In addition, food production, distribution, cost, and access in the concerned country or region are also crucial in the development process, as well as salient food beliefs, preferences, culinary practices, gastronomic culture, and other relevant factors among the people for which they are intended [12,13]. In fact, for better adherence in the case of Spain, it is critical to consider the food production structure as a Mediterranean country with genuine food traditions but also with food diversity and a rich variety of gastronomic cultures within the country [13,14]. 

The concept of FBDGs, as promoted by the FAO/World Health Organization (WHO), has existed for more than 25 years [10]; in the past, disease prevention and nutrient intake recommendations dominated the process of establishing dietary recommendations [15]. Currently, FBDGs are recognized as important tools for nutrition policy [10,11,12,13]. Promoting sustainable diets through FBDGs is part of the strategy to achieve the Sustainable Development Goals [13]. To date, a few countries have adopted more holistic approaches to FBDGs, including messages addressing food combinations as meals, eating patterns, food safety issues, lifestyles, and sustainability aspects in their FBDGs [13]. Furthermore, in recent years, several countries have developed guidelines to promote and protect traditional food cultures and consider the impact of dietary patterns and food systems on the environment [13,16,17]. 

New health challenges, societal changes, food consumption trends, and advancing scientific evidence, such as individual metabolic reactions and personalized nutrition [18], make it necessary to periodically revise and update FBDGs and that new editions include more aspects than previous ones [12,13,14,15,16,17]. Among the challenge FBDGs will need to face are environmental aspects [19] and how to provide advice for healthier nutrition transitions for increasing migrant populations from different cultural backgrounds and food habits [20]. Of note are the editorial comments that have criticized the evidence-based nature of dietary guidelines [21]. Improving trust in scientific conclusions requires implementing efficient, explicit, and reproducible methods supporting FBDGs’ development [22].

Importantly, to facilitate and improve adherence to FBDGs with a real effect on food consumption, FBDGs need to have clear links to multi-sectorial policies from a health-in-all-policies approach [10,11], particularly food policies related to school meals, hospital food service, public supply, food marketing and advertising regulations, food industry standards, etc. [10,11,13]. As clearly suggested by the WHO, countries are encouraged to integrate dietary guidelines with other health promotion policies such as physical activity, smoking cessation, or the reduction of alcohol-related harm [6,10].

Multidisciplinary interaction among professionals from different areas is key for the development of FBDGs in order to integrate knowledge covering fields such as agriculture, health, education, nutrition and food science, consumers, non-governmental organizations, the food industry, communications, and anthropology, among others [10,11,12]. The early involvement of stakeholders is recommended to gain acceptance and support [10,11,12]. 

The development of FBDGs may be carried out using a stepwise approach as suggested by FAO–WHO and the European Food Safety authority (EFSA) [10,11]: the identification of diet–health relationships; identification of country specific diet-related health patterns and problems; and disease and mortality patterns should be reviewed to identify public health and nutrition priorities and key nutrients [10,11,12,13]. Country-specific health statistics and information systems are the basics at this step [13,17]. Other sources such as the Global Burden of Disease Study [23] can be very useful as well, since it is the most comprehensive worldwide study describing mortality and morbidity from major diseases, injuries, and risk factors related to health at global, regional, and national levels. In this step, nutrient imbalances in population subgroups should be identified by thorough analysis of the best-quality evidence in the country on usual nutrient intakes and nutritional status in the population: identification of food sources relevant for FBDGs, food consumption patterns, as well as population characteristics for each pattern [10,13,17]. Recommendations in FBDGs should consider the specific needs of population groups [10,11,12,13] and be country specific [10,11,12,13,14,15,16,17]. Testing and updating FBDGs and the best way to disseminate them is also a key step in this process [10,15]. In this sense, graphical representations of FBDGs are often developed in order to facilitate communication to consumers [10,11].

Since the 1996 publication of preparation and use of FBDGs based on a joint WHO/FAO consultation [11], many countries and regions have developed their own national guidelines, often in partnership with or facilitated by international agencies and bodies [17]. Some regions have developed regional recommendations, such as the Nordic Nutrition Recommendations, which are the main basis for national FBDGs in Denmark, Finland, Iceland, Norway, and Sweden [24]. There has been coordination on guidance for developing FBDGs in Latin America and the Caribbean [25], and in the Western Pacific region [26]. However, the attempt for a unique set of European FBDGs has been unsuccessful until now. 

## 3. The Contribution of the Spanish Society of Community Nutrition to FBDGs in Spain

The Spanish Society of Community Nutrition (Sociedad Española de Nutrición Comunitaria—SENC) started at the end of 1989 with the objective of studying, developing, defining, and exchanging knowledge about community nutrition by promoting a healthy eating style based on scientific evidence. Since the very beginning, SENC considered multidisciplinary collaboration for the advancement of Nutrition and Public Health [27,28].

In 1987, in the context of the 2nd World Basque Congress, for the first time, a thorough discussion on nutritional objectives and dietary guidelines was organized in the country involving relevant Spanish experts in the field, as well as those from the UK, USA, and other countries [29]. In previous years, academic experts had prepared recommended nutrient intakes and the Ministry of Health was implementing a program on food and nutrition education (EDALNU) to meet recommended nutrient intakes and avoid imbalances using the food wheel as a pictorial model [30].

The SENC published FBDGs for the Spanish population for the first time in 1995 [31]. The document reviewed existing evidence on the nutritional status of the population and defined national nutritional objectives and FBDGs that were graphically summarized in a Pyramid of Healthy Eating. These FBDGs were based on the “Traditional Healthy Mediterranean Diet Pyramid” designed by Oldways–Harvard–WHO as an alternative to the USDA’s original food pyramid, and published by Willett et al. [32] in the American Journal of Clinical Nutrition in 1995. Those SENC FBDGs and nutritional objectives were adapted by the WHO for its food pyramid and recommendations, considering the food consumption patterns in the Spanish population which is characterized by the wide use of olive oil, the typical content and quality of fat in the Spanish diet [33].

The second edition was released in 2001 [34]. Experts and professionals in nutrition and health sciences and experts from other scientific societies linked to nutrition and public health participated in this revision. As in the previous edition, the FBDGs were aimed at professionals in healthcare, food and nutrition, education, and others in order to be used in dissemination and educational activities related to healthy eating. They were also adapted for dissemination for the general population, and a specific pyramid for children and adolescents was designed [35,36]. 

In 2004, a new edition of the food guide was prepared in collaboration with societies composed of primary healthcare practitioners for wider dissemination and use [37]. This time, the guidelines included recommendations on purchasing food as well as safety issues at home related to food conservation and food preparation.

Following the launch of the nutrition and physical activity strategy (NAOS) by the Spanish Agency for Food Safety and Nutrition (AESAN), a food and physical activity pyramid was issued by AESAN in 2005 [38]. The image showed a combination of recommendations for healthy eating and physical activity, consistent with SENC diet recommendations. The AESAN website included the 2004 edition of the SENC FBDGs for healthy eating for further reference. The AESAN adopted the specific pictorial image for children and adolescents for the school-based PERSEO program (Programa Piloto Escolar de Referencia para la Salud y el Ejercicio contra la Obesidad) [36].

In 2011, SENC published a new technical report on the nutritional objectives for the Spanish population [39]. This document fueled the process of updating the FBDGs, discussed in the context of the 3rd World Congress of Nutrition and Public Health held in Las Palmas de Gran Canaria in November 2014. The working groups conducted the evidence review during the first two years of the project; the technical report, as well as the new food guide pyramid were published early in 2017 [40]. This last edition introduced important changes compared to the previous ones. In addition to recommendations on food, it also includes aspects related to lifestyles, sustainability, emphasizes mindful eating, energy balance, maintaining traditional patterns, and stress awareness around food and eating. The new FBDGs encourage consumption of seasonal foods, underline special consideration for locally grown products, advise on the use of healthy culinary techniques, promote reduced food waste, and suggest seeking advice from an appropriate healthcare professional before using dietary and nutritional supplements.

Moreover, SENC published for the first time in 2009 the Pyramid of Hydration [41], updated in 2016, and included in the technical document supporting the current FBDGs [40].

Finally, between 2016 and 2018, SENC, in collaboration with the scientific societies composed of primary healthcare professionals, developed practical guidelines for healthy eating to convey to the population and health professionals the contents of the FBDGs with affordable and practical messages [42]. They include the Pyramid of Healthy Eating, recommended serving sizes, practical recommendations for designing menus, purchasing, storing and cooking food, specific advice for different groups of population and food education in the home. They offer particularly practical recommendations that contribute to food sustainability, such as the preferential consumption of foods that have a lower environmental impact (vegetables), are seasonal, and locally produced, as well as reduce food waste and encourage recycling.

This document was prepared by SENC in collaboration with all the scientific societies composed of primary healthcare professionals for the first time in the country, i.e., SEMG (Spanish Society of General and Family Doctors), SEMERGEN (Spanish Society of Primary Care Physicians), SEPEAP (Spanish Society of out-of-hospital Pediatrics and Primary Care), and SEMFYC (Spanish Society of Family and Community Medicine). The main objective of the document is to support the care work and nutrition education carried out by different health professionals (doctors, specialists, and nurses in primary care health centers, pharmacists, assistants in pharmacy offices, dietitians, nutritionists, midwives, physiotherapists, and podiatrists in their clinics and public or private health centers). The document can also be used as a reference for nutrition education for consumer organizations, citizen groups, and, especially, teachers at all levels of education. Also, it is a guide in general for those individuals concerned about their health. 

Over the last decade, two additional sets of diet and lifestyle recommendations have been released in Spain, those by the Mediterranean Diet Foundation [43] and the proposal by the Iberoamerican Nutrition Foundation (FINUT), including advice for healthy eating, physical activity, and hygiene [44]. Dietary recommendations in these two sets align with those by SENC. Unlike in other countries, to date, neither the Spanish Ministry of Health nor the Spanish Ministry of Agriculture have promoted participatory multi-sectorial processes for the development of scientific-based agreed upon FBDGs. The SENC FBDGs are the recommendations most widely used in Spain by healthcare practitioners, consumers, educators, and the food industry, since the publication of the first edition.

A recent review of current (FBDG) analyzed the existing guidelines in 90 countries, the earliest issued in 1986, 33 of those in European countries. A number of recommendations are consistent in nearly all sets: consume a variety of foods, some foods in higher proportion than others; consume fruits, vegetables, and legumes; and limit sugar intake. However, guidelines regarding dairy, red meat, fats and oils, and nuts are more variable, despite the WHO’s global guidance encouraging the consumption of nuts, whole grains, and healthy fats [17]. 

## 4. New Food-Based Dietary Guidelines for the Spanish Population

The new dietary guidelines (FBDG) for the Spanish population [40,42] were developed and agreed upon in a collaborative effort over the course of several years, from 2014–2018, involving an extensive group of nutrition and healthcare professionals with experience in this field and related areas. During the first two years of the project, the multidisciplinary group of experts convened by SENC reviewed the evidence about mortality and disease patterns in the country including inequalities; diet, lifestyles, and health; current nutrient and diet intake from food consumption survey data; food patterns and distribution in population groups; food preferences; culinary practices; food purchases and cost, as well as the available information on the environmental impact of the diet in Spain [45,46]. The review informed the recommendations and the pictorial icon, tested by means of group interviews. In the following two years, the primary healthcare expert group together with the SENC working group adapted the recommendations and developed an easy-to-understand friendly guide [42]. The healthcare, scientific, and educational communities and the public in general were made aware of the FBDGs in a series of sessions organized in the different Spanish autonomous regions, beginning in December 2018; the last one to date, in October 2019. 

Evidence shows that nutrition and physical activity can play a major role in curbing the burden of chronic disease [1,2,6,47]. However, primary healthcare clinicians have limited time available and lack of specific training in medical schools and in service ongoing opportunities to be confident in providing nutrition and lifestyle advice in everyday practice [47,48], which precludes sufficient emphasis on nutrition and physical activity in their interactions with patients [48].

This edition supports the idea that primary healthcare, as well as different community settings, play a key role in the implementation and reinforcement of FBDGs. Obviously strategies targeted to individuals and families are essential for the adoption of FBDGs. “Healthy Dietary Guidelines for primary healthcare and citizen groups” was edited independently by SENC through the Planeta publisher [42].

The content of the FBDGs was divided into 16 chapters and some additional annexes. The arrangement of the content pursues the route consumers follow to complete the feeding process. It starts from planning or thinking about choices for the daily or weekly menu at home and, then, follows with food purchase, food preparation, and safe preservation of food and beverages, as well as safe procedures in the kitchen (avoiding food poisoning and preventing accidents). The guide highlights useful recommendations to minimize the environmental impact of diets and tips to reduce waste, close to zero residues throughout the contents (Table 1).

The suggested framework sought for a balance between the scientific evidence and achievable changes in the short- and medium-term in the main target population, technically healthy population. The proposal suggests recommendations that are midway towards healthier eating practices within an improvement interval, so that is likely to have a positive response in practice, at the individual and group level. The advice largely respects the uses and customs of the traditional cuisine (Mediterranean diet), cooking techniques, gastronomic culture, and felt needs in relation to food, organoleptic sensations, and social relations, considerations which are so important for Spanish people [13,14,17,43]. It involved articulating a list of recommendations that, considering current practices, allowed for steps moving forward towards healthier diets and which are relatively simple to practice. The recommendations tried to avoid creating dietary advice that people could perceive as a limitation or imposition on individual freedom of choice or in disagreement with the cultural context [49,50,51]. This is a proposal for a comfortable path that contributes to building healthier and more sustainable food patterns [49,52] and recovering a traditional diet closer to the Mediterranean diet—a dietary pattern culturally sensible and affordable for many people in the country [53,54,55], although supportive policies for implementation that consider inequalities will be required. The Mediterranean diet can be considered as the most studied and well-known dietary pattern in the world [56]. A growing body of evidence supports this dietary pattern as a healthy and sustainable dietary pattern [56,57,58,59,60,61,62,63,64,65,66,67,68], particularly the PREDIMED trial (Prevención con Dieta Mediterránea) [69,70,71,72]. 

As in previous editions [31,34], the new FBDGs continue to have a pyramid as the pictorial icon, where food is located within the perspective of proportionality and health impact by means of a background color code, position in the different strata, and comments on recommended frequency of consumption. The organization of the different layers was framed considering the Mediterranean diet as the reference dietary pattern [43], consistent with the prevailing culture in Spain and its food habits and culinary practices. An orange, dotted line delimits the two bottom layers including the cereal group, starchy vegetables, fruit, vegetables, and olive oil as the food groups to include in each main eating occasion. The proportion among the layers represents the different contribution to the total energy intake by the different food groups.

On the other hand, this new version of the pyramid incorporates clear messages regarding different determinants of food intake, criteria for sustainable diets, suggestions for seeing an appropriate healthcare professional for advice on the use of food or nutritional supplements [73], and limitations on the consumption of low-grade fermented alcoholic beverages [74]. Both the posters and the FBDGs provide a table with suggestions on the recommended portion sizes according to age and sex, considering as a general reference, the usual intake in our population and the average theoretical horizon of 2000 kcal/person/day. Specific analysis of the dietary and nutrient intake in the Spanish population was conducted, as well as an analysis of the prevailing dietary patterns. Additional existing information on the usual portion sizes commonly consumed by different population groups in Spain [75] was analyzed as well. All this data were the basis for informing the suggested portion sizes in line with the recommended dietary changes. In addition, the information available on food purchases and cost was reviewed [54,55,76,77].

**Figure 1:**
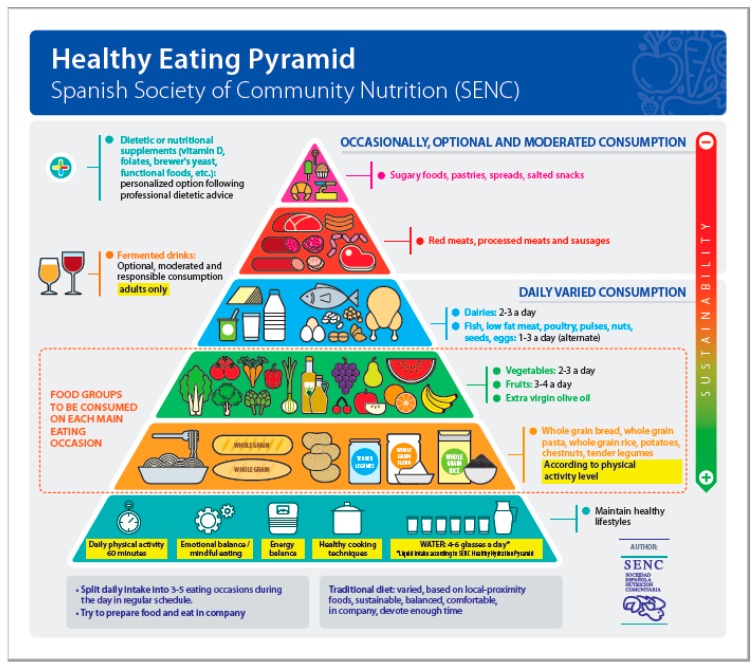
Healthy Eating Pyramid, Spanish Society of Community Nutrition (SENC).

From left to right, at the base of the pyramid, the first image reflects the recommendation to be active and undertake physical activity daily, as shown in Figure 1. The text of the FBDGs advise at least 60 min a day or its equivalent to 10,000 steps, with varying degrees of intensity according to age, sex, and health status [78,79,80]. The next icon refers to mindful eating and emotional balance as a determinant related to an adequate diet, but also as a conditioning factor when buying, cooking, and eating food. Emotional and disinhibited eating have been reported as important domains of mindful eating relevant to serving size and snacking behaviors [81,82,83]. Mindful eating consists of making conscious food choices, developing an awareness of physical versus psychological hunger and satiety cues [82], and eating healthfully in response to those cues [83]. A recent systematic review concluded that mindful eating could be a practical approach to weight control [84]. 

It has been suggested that high-calorie fatty foods, which are readily and cheaply available, are easily overindulged [85]. Integration of food-related sensory signals, (i.e., sight, smell, taste, and texture) with various peripheral homeostatic hunger/satiety-related signals, modulate the representation of the “affective value” of food, reflecting the rewarding or pleasurable aspect of foods [85,86]. Moreover, recent evidence suggests a likely impact of gut–brain signals on reward and emotion [85,87] which occurs independently of sensory properties, such as taste. It has been described that people who report higher levels of perceived stress and time pressure tend to be more convenience-oriented in relation to food purchases [88,89] and more likely to consume fast foods on a regular basis. Impulse buying is a set of automatic behaviors driven by heuristic processes which represent the interplay between intra-individual psychological factors and environmental cues [90,91]. Research shows that impulsive consumers tend to make food choices based on taste preferences [91], and increased impulsivity is associated with a stronger tendency to overeat in response to external food cues [91,92].

In addition, there are suggestions to consider energy balance [93,94] in order to maintain body weight within the healthy range: move more when we eat more considering the qualitative and quantitative nuances at both sides of the equation—intake, physical activity, and paying more attention to a healthier diet while limiting portion sizes if we move less. Important remarks related to healthier culinary techniques are included as well.

**Figure 2:**
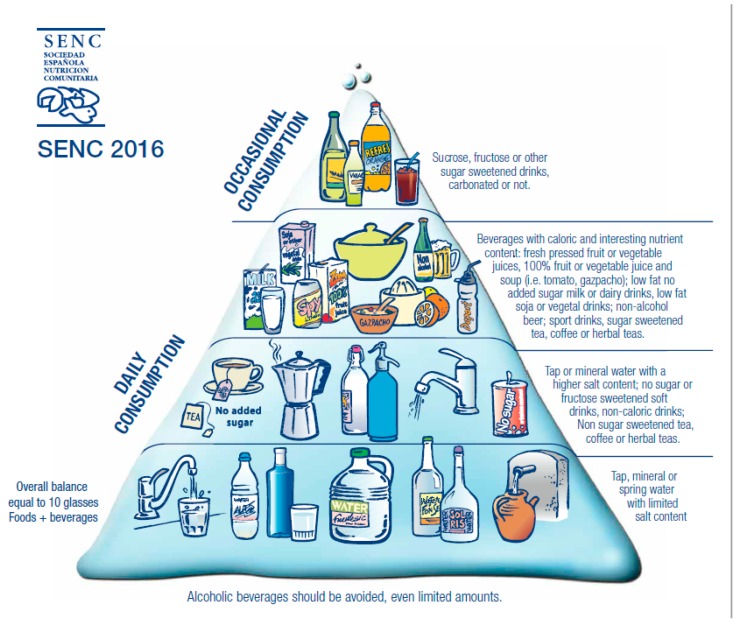
Healthy Hydration Pyramid, Spanish Society of Community Nutrition (SENC).

The base is completed with a jug and glasses of water that advise on ensuring adequate fluid intake for an optimal hydration status as fundamental to achieving and maintaining health and well-being [10,95]. Figure 2 shows the latest proposal for the Healthy Hydration Pyramid [40]. The Pyramid of Hydration includes advice for healthy hydration in the context of a healthy diet. This pyramid was designed considering water intake levels in different population groups, sources, and contribution of beverage consumption to energy and nutrient intake in addition to their contribution to water intake. The current version of the pyramid is a modification of the previous one following the update of the FBDG based on updated consumption information. The general basic recommendation encourages the consumption of water, tap water wherever available, that is safe to drink and palatable. Recommendations regarding other beverages consider their contribution to energy and nutrient intake as well as to water intake in light of the current consumption practices in the population. Thus, the first two levels mainly suggest water of varying mineralization grades and then different beverages without caloric content, as a priority for proper hydration.

Beverages with high water content and nutritional value include fresh-pressed fruit juice, vegetable juices, and soups such as “gazpacho”, milk, vegetable drinks, etc. This group of beverages supplies high water content, but also a varying contribution to the intake of nutrients such as vitamins and minerals. However, they may also contain added sugar, salt or fats. Sport drinks are increasingly consumed to replace conventional soft drinks for the lower sugar content, particularly among adolescents and young people in Spain [96,97]. This group of beverages is located in the third level of the pyramid. In the upper step, with a recommendation for optional and occasional consumption, are located beverages with added sugars or similar components, and especially those with high sugar content. Below the bottom of the pyramid, there is a text box flagging that alcoholic beverages do not contribute to hydration and should not be used with this function. Alcoholic beverages do not contribute to hydration [98].

Following the food pyramid, the second and third levels incorporate the foods characteristic of the Mediterranean diet [43], framed together (orange, dotted line) as the basic food groups to be included in each main eating occasion. Firstly, the image highlights whole grain cereals and the wide range of related products (bread, pasta, and flours to make different products) [99,100,101,102], together with potatoes, chestnuts, and fresh legumes (peas, beans, fresh beans, etc.). Then, on the following level, are included all kind of fruits (three portions per day), vegetables (three portions per day), and aromatic herbs [102,103,104]. At this level is located extra virgin olive oil (EVOO) as well, with a preference for cold-pressure EVOO [105,106].

Low-fat dairy products (fresh cheeses, natural yogurt, milk without added sugar) (2–3 portions per day) [107,108,109] and a section including white meats, eggs, dried legumes, nuts, and fish are [110,111,112,113,114] located immediately above, with a recommendation to include 1–3 portions per day of this groups, alternating between groups. The text adds comments to create awareness on priorities about modes of livestock breeding [115], seasonality, sustainable fish captures [116], and the recommendation for moderation in the consumption of protein from animal sources and encouragement of the consumption of protein from plant and alternative sources, with lower environmental impact [117]. Recommendations in this regard considered recent analyses conducted in the country on the environmental impact of dietary patterns in Spain [45,46,68,72,117]. This is the last block of foods with potential recommendations for daily consumption, according to seasonality and preferences.

The upper level, with a label for an optional and much more occasional and moderate consumption, include red meat, sausages, and similar products. It is advised to reduce the consumption of foods from this block [118], consume lower amounts, and to show interest for better quality products, considering the feeding and rearing of livestock as well as the environmental footprint [115].

At the very top of the image, in the vertex, are located foods advised for optional consumption, and, if so, in very small amounts, (i.e., foods with high-fat, salt, and/or sugar content, ultra-processed products, goodies, sweets, and industrial pastries [119,120]).

The pyramid on its lateral cusp displays a warning message to consider the need, usefulness, and caution about the consumption of special foods, herbs, pharmacological supplements, diet and nutrition supplements, nutraceuticals, and the like [73]. A cross with four colors recalls the need to always consult and seek advice from healthcare professionals (doctor, pharmacist, nurse practitioners, and dietitians-nutritionists) about the consumption, dose, duration, potential interactions, and changes in food intake that would make their consumption unnecessary and other suitable considerations [121,122]. Consumers, patients, and healthcare professionals should critically assess whether the addition of supplements to a conventional diet is needed or not depending on clinical or personal circumstances, and, if required, value relevant biomarkers to assess possible deficits or suboptimal values (vitamin D, folates, vitamin B12, iron, etc.) likely to improve [73,121,122].

Consumption of alcoholic beverages is a controversial issue. The guidelines do not encourage or recommend alcohol consumption for health, but acknowledge the fact that a high proportion of citizens use alcoholic beverages on a daily basis [123,124]. The advice reminds that the only evidence supporting alcoholic beverages is bounded in the context of the Mediterranean diet to limited amounts of low-grade fermented alcoholic beverages (wine, beer) with meals [125,126]. Taking into account the prevalence of the Mediterranean culture in the country, the uses and customs of the population, and the scientific evidence available [123,124,125,126,127], we deemed appropriate to adopt the following position:

- People who do not use alcoholic beverages should not start drinking because of the potential beneficial effects attributed to low-grade fermented alcoholic beverages. An optimal diet can achieve equivalent results without the potential risks of drinking alcoholic beverages, even if they have low alcohol content.
- Drinks that can be considered are limited exclusively to low-grade fermented alcoholic beverages (wine, beer, cider, cava, or champagne (sparkling wines with double fermentation)).
- The evidence supporting a beneficial effect of consumption refers to alcohol consumption in the context of the Mediterranean diet, with meals, and in very moderate amounts (15 g alcohol/day) [125,126].
- Such tolerance refers exclusively to adults with a consumption limited to no more than 2 glasses of wine or equivalent (200 mL; <40 g alcohol/day) for men and no more than 1 glass for women (100 mL; <20 g alcohol/day) [125,126].
- People with chronic diseases or taking medications or supplements should consult with a doctor and follow recommendations at all times, even if advised to avoid alcohol altogether.
- Spirits and liquor should be avoided in the context of a healthy diet and lifestyle.
- In Spain, there are good-tasting, socially acceptable alternatives for private or social consumption of alcoholic beverages, such as non-alcoholic beer, de-alcoholized wines, and similar products available in virtually all establishments in the country.
- Regarding the location of fermented beverages external to the main content of the Healthy Eating Pyramid and the specific section in the FBDGs, the decisive factor was that, in Spain, wine is legally considered “food” (Ministry of Agriculture, Fisheries and Food), aside from its important cultural, gastronomic, economic, and social value. Excluding this reference and similar beverages from the graphical representation or from the contents of the FBDGs would not per se prevent excessive or inappropriate consumption and, most importantly, it would mean that the Guide could not clearly provide criteria and limits for consumption or its reasoned exclusion.

**Figure 3:**
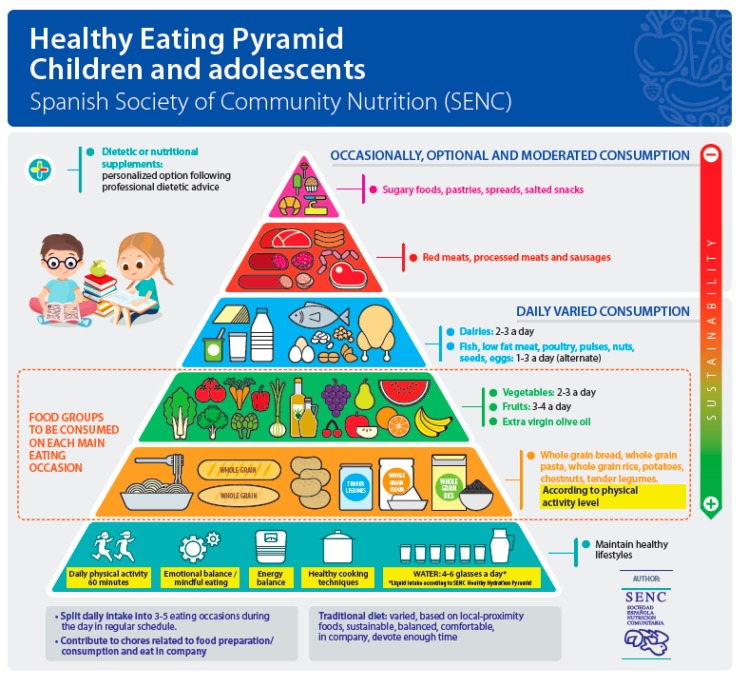
Healthy Eating Pyramid for children and adolescents, Spanish Society of Community Nutrition (SENC).

The pyramid has been translated into the three co-official languages in Spain (Basque, Catalan, Galician) along with summaries of the contents of the FBDGs to facilitate use and comprehension in the corresponding autonomous regions. A differentiated version has also been presented for minors, where the section of fermented beverages has been replaced by a study vignette to represent “pre-school–high-school periods of life”. The physical activity symbol represents an active family together (Figure 3).

On the outside of the pyramid, at the bottom zone, three labels remind of the interest of splitting up intake [128], the importance of cooking and eating with company, devoting sufficient time [43,129,130], and a clear recommendation to prioritize the consumption of seasonal, local, and sustainable food [131].

The design of the pyramid required an intense creative activity entangling different health professionals linked to SENC and the other societies involved, design experts, and members of the public. Eight group discussions (*n* = 64) were organized for the validation of the pyramid. Participants in the validation and adjustment phase included healthcare professionals, students of the last academic term of the Degree in Nutrition and Dietetics, teaching professionals, and consumers. Consumers commented on the comprehension of the picture, how they felt about it, sources of information about healthy nutrition, and discussion on changes they were aware of having introduced into their diets following messages in the media.

### 4.1. Collaboration with the Scientific Societies of Family and Community Medicine 

One of the strengths of the new version of the FBDGs was the involvement of the coordinators of the nutrition committees of the four scientific societies representing primary healthcare in Spain in a collaborative group. Three of them are related to adult healthcare and one to children and adolescent care (from birth to 14 years.) (SEPEAP). This fact facilitates the following operational points:

1. Its use in primary healthcare centers and clinics in public and private healthcare systems, both in healthcare practice and in health promotion programs;
2. Stimulation of brief dietary advice in healthcare practice, whenever deemed suitable;
3. Bolsters healthcare practitioners to address the demands of information by patients with greater confidence in the proposed recommendations;
4. Helps healthcare professionals to feel confident because they have updated, agreed upon, approved, and homogeneous criteria in hand for application and points of reference (health center, pharmacy, nursing or diet and nutrition consultation);
5. Enables its use as a resource in educational settings and other community groups in information and health education actions.

### 4.2. New Sections: Diet in the 21st Century

The FBDGs is fully committed to food sustainability and all associated processes; this is one of the backbones of the recommendations inside and outside the home. There is a chapter dedicated specifically to this topic and the message is a priority across all the contents. Table 2 summarizes the decalogue for sustainable diets in the community, appended as an annex [131]. 

A section is devoted to commenting on the interest of precision nutrition based on the advances in all “omic” sciences [18,132] and, also, on the better understanding of the role played by microbiota and the intestinal microbiome in health and disease [133]. An individual’s response to food intake and its components results from the interaction of metabolic, genetic, environmental, and social factors [16]. “Personalized nutrition” refers to the adaptation of the diet to individual needs and preferences. “Precision nutrition” predicts whether a given subject will respond to certain nutrients and dietary patterns so that it can contribute to disease prevention [134]. This chapter includes a warning message about possible fraud, the need for precaution in the face of genetic tests and advice on consulting the family doctor or specialist about the suitability of this type of resource or of a specific application.

We were especially interested in the need to establish criteria for generalized pre-conception dietary advice that, together with other tests or recommendations, could be useful for men and women wishing to have children, with a greater commitment to their health. This chapter is followed by a series of sections on diet at different stages of the life cycle.

One chapter is concerned and addresses nutrition education in different environments: schools, healthcare, and occupational settings. Diet 3.0 and new apps linked to information technologies deserve attention as well, including trusted sources of information related to food, nutrition, and health. Navigating the internet to search for food- and health-related information retrieves information from all kind of sources. Often the first results correspond to advertising messages with commercial interests. This kind of information should be searched for on sites that offer reliable information [135,136]. It is also necessary to have tools that allow to easily recognize when a website offers reliable information. To this end, different organizations have created quality labels that identify web pages with quality information [137]. People are encouraged to check the information with healthcare professionals before making any decision about health.

A comprehensive chapter is dedicated to dietary recommendations for the most prevalent ill health conditions and risk factors managed in primary healthcare. In addition, a broad annex of drug–nutrient interactions is included for consideration by doctors, specialists, and community pharmacists [138]. Sometimes there may be a potentiation or inhibition of the effect of certain medications depending on the simultaneous consumption of some foods or beverages (alcohol, grapefruit juice, fiber, etc.). While some medications may be more appropriate to consume before meals, in other occasions it may be better to take them during meals or after meals. There may also be an interference among different medications and even among different nutrients or dietary supplements [139]. People are advised to read carefully the leaflets of medications and, in case of doubt, consult a doctor and/or pharmacist.

The last section of the FBDGs is devoted to the chronobiology of nutrition and verified applications. There is a clear relationship between alteration of the circadian system and metabolic syndrome, alteration of cholesterol metabolism, diabetes, and cardiovascular diseases [128]. Shorter sleep time, jet lag caused by long-distance travel, shift work, increased exposure to bright light at night, high consumption of snacks during the day and night, changes in the hours of lunch and dinner, etc., are factors that can modify the perception of internal and external rhythms [140]. Based on existing evidence, it would be advisable to have dinner at least two and a half hours before going to bed, avoid eating sweets, sugars or cakes before going to bed, and avoid intense physical activity 2–3 h before the usual sleep time [140]. Finally, the guide contains annexes with a bibliography for further reading and complementary information sources.

In summary, this is a document including updated recommendations on diet, physical activity, and other practices relevant to health and nutrition presented in a friendly and easy-to-understand format for healthcare practitioners, consumers, and others. The recommendations are reasonably affordable for the average citizen to implement in a sustainable and comfortable way, as well as in family and community environments when following the principles and bidirectional strategies of action posed by community nutrition [141,142]. For that purpose, each chapter has an informative summary with specific indications for its application in daily practice.

## 5. Discussion

Food-based dietary guidelines are useful tools to convey clear and easy-to-understand messages to a wide audience, with the aim to facilitate the adoption of healthier dietary patterns [10,11] based on the best available evidence. As the body of evidence increases, social, food consumption, and eating practices will also change under the influence of multiple factors. This makes it necessary to periodically review and update FBDGs so that they are operational and responsive to changing circumstances [10,11,13,17,52].

In addition, the paradigms inspiring policy and practice in public health nutrition have changed over the years. Although different approaches coexist, current reviews emphasize selected food patterns [49,143,144,145]. Growing evidence suggests that eating habits and practices tend to cluster into dietary patterns, which more or less overlap with specific a priori defined patterns [146]. Moreover, a number of studies also suggest that food patterns tend to coexist with other lifestyles [147,148].

Evidence suggests that greater adherence to the Mediterranean diet contributes to reduced risk factors for cardiovascular diseases (CVDs), some types of cancer, metabolic and degenerative problems, cognitive decline, depression, and foster longer life expectancy with better quality of life [56,57,58,59,60,61,62,63,64,65,66,67,68,69,70,71,149,150]. The evidence supports as well that other food patterns, such as the DASH diet (Dietary Approaches to Stop Hypertension) or the new Nordic diet, can also reduce the risk for a number of health problems [151,152]. All these patterns share peculiarities, such as a high consumption of fruits and vegetables, fish, and wholegrains, along with moderate-to-low consumption of red meats, saturated fats, ultra-processed foods high in salt and/or sugar, and sugary drinks [68,69,70,71,144,145].

In fact, the latest edition of the USDA dietary guidelines for Americans [145], as well as those released in Australia [143], focus attention on food patterns that meet these characteristics. As noted, especially since the recognition of the Mediterranean Diet as a UNESCO Intangible Heritage of Humanity [153], the concept extends beyond a set of food and beverages consumed in certain proportions. The vision of the Mediterranean diet implies a dietary pattern based on seasonal products of the territory, linked to traditions, culinary culture, and other dimensions related to food procurement, preparation, and consumption [5,43]. Conviviality around food and eating are central to traditions and everyday life [154]. Furthermore, key ingredients of the traditional Mediterranean diets shape the countryside, as they are locally produced. However, the impact of globalization and progressive abandonment of rural areas towards urban locations over the past decades, along with the increasing industrialization of food production, distribution, and even food preparation have led average diets to deviate from traditional Mediterranean diets, particularly among children and adolescents [155,156,157].

The SENC signed and supported the call to act and revitalize the Mediterranean diet launched in July 2016 in Milan [5]. The SENC FBDGs promote the traditional Mediterranean diet and advocate for the slow way of life, devoting adequate time to purchasing food, preparing food, and eating together. Furthermore, it attempts to persuade consumers to buy locally grown, seasonal foods whenever possible, encourage people to inquire about the way foods are produced, and demand sustainable food products, from producers to the table, thus caring about food waste, recycling, and reduction of packaging and related waste. 

Tapsell et al. [49] recommended that the collection of evidence should follow a systematic top-down approach, starting with dietary patterns, followed by single food groups and then by nutrients. Fruits, vegetables, cereals, fish, legumes, dairy products, and nuts are the key ingredients of the Mediterranean diet, along with moderate intake of meats, especially lean meats, seasoned with mainly good quality olive oil, and accompanied with moderate consumption of wine during meals [43]. In line with this, in this update of the FBDGs for the Spanish population, the benefits of such a pattern are recognized and the recommendations are based on the main features of the Mediterranean diet, while being aware of current prevalent uses and customs in the country, as well as the culinary and cultural wealth of the different autonomous regions. This recommendation is aligned with those in most FBDGs, particularly in European countries, as noted in recent reviews and comparative analyses of FBDGs in the world [13,16,17,158].

This set of FBDGs holds a holistic vision of food systems, in tune with the territory, the environment and sustainability, and, at the same time, inspired by traditions and modeled by the social reality of the moment. In this sense, we consider that emotions, perceived well-being, and stress influence food choices [90,91,159], as well as attitudes and behaviors related to food and drink purchases, preparation, and consumption [160]. Diet quality has been independently associated with education level, food cravings, and awareness towards eating [81]. Emotional and disinhibited eating have been reported as important domains of mindful eating relevant to serving size and snacking behaviors [82,83]. Thus, FBDGs support the need to focus attention on food choices and mindful eating, as well as to devoting adequate time to buying, preparing, and eating food with company whenever possible [81,83]. 

One of the most striking and often controversial components related to FBDGs is the graphic icon to illustrate the key messages. In the past decades, the models based on the nutritional composition of the different food groups inspired food wheels in order to facilitate food choices to avoid nutritional deficit [30,158]. New pictorial representations emerged in the late 1980s in the form of a pyramid. This type of icon aims to reflect the idea of the proportionality among different foods in the usual diet, placing foods at different levels, depending on the recommended frequency of consumption from daily (base) to occasionally (top) [13,17].

More recently, in the 21st century, the plate model was suggested [13,17]. This model is intended to represent the proportion of the main food groups in the daily diet. Other countries have proposed graphic models based on cultural traditions that better focus on the target population, such as clay pots in Guatemala or Bolivia, pagodas in China and other countries in Southeast Asia, and the eastern flag in Korea [158]. The debate is open and not free of controversy. In fact, some countries have recently published FBDGs without a pictorial representation, such as Nordic countries, Chile, and Brazil. The FAO includes an extensive worldwide compilation of FBDGs and pictorial icons. Interestingly, recent reviews have analyzed differences in the pictorial icons used considering the shape, the number of food groups, proportionality, and color shading [13,17,18,158].

Our proposal continues with the pyramid model used in previous editions, representing usual foods familiar to everybody. In the Spanish cultural and social context, main meals usually consist of a sequence of three dishes, rather than placing all foods together on one plate at a time. The plate model that combines different foods on one plate may not be fully understandable. In addition, serving all foods together on one plate is associated with the concept of a ‘’combination plate’’, a sort of fast food usually based on ingredients that would not be typically located at the base of the pyramid. Furthermore, with the pyramid model proposed, in addition to the idea of proportionality in the presence of different food groups, we also want to reinforce the idea of alternation and variety. The image stresses the recommendation to configure a daily diet with the basic components, located in the block delimited by the orange, dotted line, which frames the key foods in the Mediterranean diet and the food groups to be consumed on each main eating occasion.

In the upper part of the pyramid are those foods that, due to the fact of their high content in saturated fats, sugars and/or salt, and processed foods, their usual consumption as part of a healthy diet is not recommended. However, an important premise in this process of preparing dietary guidelines is to recognize as a starting point the eating habits of the population [10,11,50]. Therefore, a proposal for a written recommendation is not included, but both the recommendations and the image highlight that foods included in this block can be omitted; consumption of this group is optional and, in any case, when consumed, intake should be occasional and in limited amounts.

In the context of the Mediterranean diet, the consumption of wine and low-grade fermented alcoholic beverages is recognized in moderate amounts during meals [125,126]. This proposal of FBDGs does not recommend in any case the consumption of alcoholic beverages. However, within the reference framework, it suggests an optional consumption of wine and fermented beverages only for adults who so desire and are not subject to contraindication due to the presence of a health condition or medication use and reminds that consumption should be limited and responsible.

Although, to a lesser extent than in other countries, a considerable proportion of the population spontaneously consumes dietary and nutritional supplements and special foods including protein and other supplements promoted in certain environments of exercise training and sport activities. Evidence suggests that this spontaneous consumption is not risk-free [73,121,122]; thus, a recommendation is included to seek the advice of health professionals with specific training in nutrition to individually guide any continued intake of supplements and special products.

In this edition of the FBDGs, we want to highlight the idea of promoting sustainable diets in tune with the territory. Other reviews of FBDGs, such as those in Australia [143], Nordic countries [144], Germany, Sweden [13,16], and Brazil [161], place special emphasis on this dimension [13,16]. Countries such as France, the Netherlands, and the United Kingdom are also making efforts in this line [162]. A report published by the FAO in 2016 analyzed this and considered sustainability in FBDGs [163]. There was consistent evidence suggesting that dietary patterns with a higher content in plant-based foods (i.e., vegetables, fruit, legumes, seeds, nuts, whole grains) and lower content in animal-based foods, total energy content, and discretionary foods and beverages were healthier and associated with a lower environmental impact [19,52]. Conclusions of recent systematic reviews on recommendations in existing FBDGs and approaches used to develop FBDGS emphasize the need to advance and further contribute in this line [4,13,17].

The economic crisis that has lasted since 2008 in Spain had an impact on the food lifestyle of many people, putting many families at risk of food insecurity, especially single parent families as well as families with children with all their members unemployed. Furthermore, social inequalities in following guidelines on a healthy diet during this period of economic crisis have been described in Spain [55]. As in many other countries, Spain is facing the challenge of increasing migrant populations from different cultural backgrounds and food habits. In previous editions of the guidelines, a specific initiative was later devoted to this analysis, and adaptation of the FBDGs was supported by AESAN [164]. In this new edition, such analysis and adaptation are still pending.

In a society of abundance and waste, including food waste recommendations for healthy food will need to consider solidarity, support fair trade, urban gardens, and ethical practices in relation to agriculture, livestock, fisheries, and food distribution in order to be consistent. This is an additional challenge for new updates in FBDGs. 

Recent systematic reviews and editorial comments suggest that organizations that produce FBDGs do not adhere to internationally recognized standards in analyzing the evidence to produce evidence-based recommendations [21,22]. In order to minimize bias and gain independence, a potential solution suggested is the collaboration with independent groups with content expertise and skills in the methodology of systematic reviews and practice guidelines to produce trustworthy guideline recommendations [165]. 

The recommendations in FBDGs are addressed to the general “healthy” population. People with acute health problems, chronic diseases or risk factors related to diet or other lifestyles, frail elderly people, or people with special needs, will need individualized adjustments in quantitative, qualitative aspects, the use of supplements, special foods or nutritional assistance in a professional healthcare context [166].

Finally, a dissemination plan, strategies, and policies that favor implementation of FBDGs are essential to support adoption and dietary change [10,11]. As part of this plan, SENC has been intensively working over a two-year period in collaboration with the scientific societies gathering health professionals involved in primary healthcare practice, i.e., SEMERGEN, SEMFYC, SEMG, and SEPEAP. A product of this collaboration was the release of an attractive, colorful, easy-to-understand publication to provide guidance and support for dietary advice and nutrition education in primary healthcare, community health, and health promotion actions. 

It is important to note that the healthcare system in Spain, although decentralized, shares common principles, such as universal coverage. Thus, healthcare centers are the reference centers in the provision of primary healthcare, involving GPs, pediatricians, nurses, social workers, and other health professionals. Regarding primary healthcare in Spain, competencies are not limited to the provision of care to people demanding assistance. They also include follow-up for people with chronic diseases and risk factors and elderly and frail people, as well as health screenings and follow-up of healthy people, and participation in community health as well as in health promotion actions. 

In particular, pediatricians and nurses in departments of pediatrics in primary healthcare centers are responsible for the follow-up of health, growth, and development of children and adolescents from birth up to 14 years of age. In addition, they are often involved in the support and implementation of health promotion actions in schools. Therefore, involving all those health professionals in the implementation of FBDGs seems to be a cornerstone for a successful dissemination and adoption. 

Furthermore, pharmacists also play an important role in providing health and dietary advice to the population. There are over 22,000 pharmacies scattered throughout the country. Specially, pharmacists active in community pharmacy collaborate on health promotion and community health actions. Thus, SENC has also developed an agreement for collaboration with this professional group to create synergies in the dissemination of FBDGs and in the development of future collaborative actions.

Among the limitations in the process followed by the SENC working group for the development of FBDGs for the Spanish population, we can mention that the expertise in the group did not include areas such as agriculture or economy. However, the range of experts involved has increased from the first FBDGs produced by SENC through subsequent updates. Another salient limitation is that no governmental body initiated the process. Nevertheless, SENC was the first group committed to follow a multidisciplinary, stepwise process to develop FBDGs in Spain, which is consolidated. The SENC FBDGS are widely used. The fact that a multidisciplinary approach was already considered in the first edition of the FBDGs is one of the strengths of the process. Another strength is the involvement of primary healthcare practitioners in the developmental phase of the FBDGs. The strategy contributes to wider dissemination, support, and adoption. In addition, the process included qualitative evaluation of the pictorial summary. This feedback help to better adapt the recommendations. Moreover, sustainability is a priority of the guidelines.

## 6. Conclusions

Food-based dietary guidelines are useful tools for preventive actions and health promotion and are helpful for guidance in health, education, and community settings.

The contents and recommendations in FBDGs must be in tune with the actual food, cultural, gastronomic, and even economic context in order to propose changes in usual diets that people can comfortably afford in the short and medium term, with the idea that it can be maintained over time.

Food-based dietary guidelines are a set of suggestions and recommendations for the general population or for specific population groups. People can follow and maintain dietary advice and dietary prescription (which may imply important changes in the usual diet) for a longer or shorter period if motivated within the framework of professional, personalized, and justified practice.

In the case of using a representative pictorial icon of the recommendations, this should be familiar, easily understandable, and reflect the food reality of the country and prevalent eating habits.

The new FBDGs must decisively consider the key aspects of food sustainability, food transitions linked to migration, food ethics, fair trade, and the humanization of the entire livestock breeding and production processes. Consumers are an essential link for change.

The health administration must support and take on these initiatives independently originated from the scientific community and scientific societies, considering their foundations in food and health policies. The food industry must also see an opportunity for improvement, reformulation, and implementation of best practices in food production and food processing, as well as in the provision of related services.

## Figures and Tables

**Table 1:** Sections and contents of the healthy eating guide for healthcare professionals and citizen groups (SENC).

1. Dietary recommendations: How to plan a healthy diet
2. Designing a daily and weekly menu that is balanced, appealing, and healthy
3. Guidance for intelligent, healthy, and sustainable food purchases
4. How to read nutrition labels
5. Cooking and food preparation processes: safe and healthy cooking techniques
6. Preservation and reconstitution of foods at home
7. 10 tips for safety in the kitchen
8. How to better manage food waste (food Rs): reduce waste, reuse, recycle, repair, reject, and responsibility when buying
9. Precision feeding: a new perspective
10. Diet, pregnancy, and breast-feeding: preconception dietary advice
11. Diet in childhood and youth
12. Diet in the older adult and aging processes
13. Nutrition education at home, in schools, in primary healthcare, and at work.
14. Diet 3.0
15. Dietary advice in primary healthcare: drug—nutrient interactions
16. Chronobiology of nutrition: principles and applications

**Table 2:** Decalogue for sustainable diets in the community.

1. Select and consume locally sourced foods and products grown in your region and buy them in local markets nearby.
2. Whenever possible, consume foods that are in season. They are healthier, cheaper, and sustainable.
3. Value traditional local food and recipes; they are part of our culture and make up our identity.
4. Learn to buy and cook in the company of others, it is more fun and enriching and we learn from each other.
5. Plan your menu and shopping lists. Try to reduce food waste and recycle adequately at home and in the community.
6. Prioritize plant-based foods. Limit the consumption of meat, processed meats, and dairy products. Your health and the planet will appreciate it.
7. Terrestrial and aquatic biodiversity is critical. We should promote it to ensure its continuity. It is everyone’s responsibility.
8. Take an interest in whether the agricultural, livestock, and fishing procedures which provide the foods you eat are SUSTAINABLE.
9. Enjoy the company and the pleasure of food at mealtimes but always within the context of balance and moderation. Reduce portion sizes.
10. Enjoy the Mediterranean diet. It is one of the best examples of a healthy and sustainable diet. UNESCO declared it as an Intangible Cultural Heritage of Humanity.

Source: Serra-Majem L (ed.). Decalogue developed in the Expert Meeting organized in collaboration with SENC in Las Palmas de Gran Canaria in April 2016 [131].

## Appendix A

Collaborative Group for the Dietary Guidelines for the Spanish population (SENC) (C.G.D.G.-SENC): Javier Aranceta-Bartrina (coord.); Victoria Arija ^9^; José Manuel Ávila-Torres ^7^; Guadalupe Blay-Cortes ^10,11^; Beatriz de Diego-Blanco ^12^; José Manuel Fernández-García ^13,14^; Marta Garaulet ^15^; María Garriga-García ^16^; Marta Gianzo-Citores ^17^; Angel Gil ^2,18,19^; Ana M. López-Sobaler; Venancio Martínez ^20,21^; Emilio Martínez de Victoria ^18,19^; Mariela Nissensohn ^2,6^; Rosa M. Ortega; Adriana Ortiz-Andreluchi ^6^; Teresa Partearroyo; Carmen Pérez-Rodrigo; Joan Quiles- Izquierdo ^22^; Lourdes Ribas-Barba ^8^; Amelia Rodríguez-Martín ^23^; Emma Ruiz-Moreno ^24,25^; Mar Ruperto-López ^5^; Gemma Salvador-Castell ^26^; Susana Santiago-Neri ^1^; Lluis Serra-Majem; Josep A. Tur ^2,27^; Teresa Valero-Gaspar ^7^; Gregorio Varela-Moreiras; Mercé Vidal-Ibáñez ^28^.

9 Faculty of Medicine and Health Sciences, Universitat Rovira i Virgili, 43201 Reus, Tarragona, Spain; victoria.arija@urv.cat
10 Grupo de Nutrición SEMG, 28005 Madrid, Spain; lupe@semg.es
11 Servicio de Salud de Aragón, 50013 Zaragoza, Spain
12 COMPASS Group Spain, 28054 Madrid, Spain; beatriz.dediego@compass-group.es
13 Coordinador del Grupo de Nutrición de SEMERGEN, 28009 Madrid, Spain; Jose.Manuel.Fernandez.Garcia@sergas.es
14 Servicio Gallego de Salud, 15970 Santiago de Compostela, Spain
15 Department of Physiology, University of Murcia, Murcia Spain; garaulet@um.es
16 Hospital Universitario Ramón y Cajal, 28034 Madrid, Spain; maria.garriga@salud.madrid.org
17 Biobanco Vasco para la Investigación-Fundación Vasca de Innovación e Investigación Sanitarias (BIOEF), 48902 Barakaldo, Spain; Martagnz_2@hotmail.com
18 Institute of Nutrition and Food Sciences, University of Granada, 18010 Granada, Spain; agil@ugr.es; emiliom@ugr.es
19 Fundación Iberoamericana de Nutrición, FINUT, 18016 Armilla Granada, Spain
20 SEPEAP, 08006 Barcelona, Spain; venancioms@telecable.es
21 Centro de Salud El Llano, Servicio Asturiano de Salud, 33209 Gijón, Spain
22 Conselleria de Sanidad Universal y Salud Pública. Generalitat Valenciana, Valencia, Spain; quiles_joa@gva.es
23 Universidad de Cádiz, 11009 Cádiz, Spain; amelia.rodriguez@uca.es
24 National Center for Epidemiology, Carlos III Institute of Health, Avda. Monforte de Lemos, 5, 28029 Madrid, Spain; e.ruiz@externos.isciii.es
25 CIBERESP (Consortium for Biomedical Research in Epidemiology and Public Health), Madrid, Spain
26 Departament de Salut, Generalitat de Catalunya, 08005 Barcelona, Spain; gemma.salvador@gencat.cat
27 Research Group on Community Nutrition & Oxidative Stress, University of the Balearic Islands, 07122 Palma de Mallorca, Spain; pep.tur@uib.es
28 Nutrición Sin Fronteras, 08029 Barcelona, Spain; merce@nutricionsinfronteras.org
